# Grip Strength, Neurocognition, and Social Functioning in People WithType-2 Diabetes Mellitus, Major Depressive Disorder, Bipolar Disorder, and Schizophrenia

**DOI:** 10.3389/fpsyg.2020.525231

**Published:** 2020-11-25

**Authors:** María Aliño-Dies, Joan Vicent Sánchez-Ortí, Patricia Correa-Ghisays, Vicent Balanzá-Martínez, Joan Vila-Francés, Gabriel Selva-Vera, Paulina Correa-Estrada, Jaume Forés-Martos, Constanza San-Martín Valenzuela, Manuel Monfort-Pañego, Rosa Ayesa-Arriola, Miguel Ruiz-Veguilla, Benedicto Crespo-Facorro, Rafael Tabarés-Seisdedos

**Affiliations:** ^1^Department of Medicine, Faculty of Medicine and Dentistry, University of Valencia, Valencia, Spain; ^2^Faculty of Psychology, University of Valencia, Valencia, Spain; ^3^TMAP – Unidad de Evaluación en Autonomía Personal, Dependencia y Trastornos Mentales Graves, University of Valencia, Valencia, Spain; ^4^Centro Investigación Biomédica en Red de Salud Mental, CIBERSAM, Madrid, Spain; ^5^INCLIVA Health Research Institute, Valencia, Spain; ^6^IDAL – Intelligent Data Analysis Laboratory, University of Valencia, Valencia, Spain; ^7^Faculty of Psychology, EAFIT University, Medellín, Colombia; ^8^Department of Physiotherapy, Faculty of Physiotherapy, University of Valencia, Valencia, Spain; ^9^Department of Physical Education Teacher Training, University of Valencia, Valencia, Spain; ^10^Department of Psychiatry, Marqués de Valdecilla University Hospital, IDIVAL, School of Medicine, University of Cantabria, Santander, Spain; ^11^Hospital Universitario Virgen del Roció-IBIS, University of Sevilla, Seville, Spain

**Keywords:** frailty, grip strength, cognitive performance, social functioning, severe mental illness, type-2 diabetes mellitus

## Abstract

Background: Frailty is a common syndrome among older adults and patients with several comorbidities. Grip strength (GS) is a representative parameter of frailty because it is a valid indicator of current and long-term physical conditions in the general population and patients with severe mental illnesses (SMIs). Physical and cognitive capacities of people with SMIs are usually impaired; however, their relationship with frailty or social functioning have not been studied to date. The current study aimed to determine if GS is a valid predictor of changes in cognitive performance and social functioning in patients with type-2 diabetes mellitus and SMIs. Methods: Assessments of social functioning, cognitive performance, and GS (measured with an electronic dynamometer) were conducted in 30 outpatients with type 2 diabetes mellitus, 35 with major depressive disorder, 42 with bipolar disorder, 30 with schizophrenia, and 28 healthy controls, twice during 1-year, follow-up period. Descriptive analyses were conducted using a one-way analysis of variance for continuous variables and the chi-squared test for categorical variables. Differences between groups for the motor, cognitive, and social variables at T1 and T2 were assessed using a one-way analysis of covariance, with sex and age as co-variates (*p* < 0.01). To test the predictive capacity of GS at baseline to explain the variance in cognitive performance and social functioning at T2, a linear regression analysis was performed (*p* < 0.05). Results: Predictive relationships were found among GS when implicated with clinical, cognitive, and social variables. These relationships explained changes in cognitive performance after one year of follow-up; the variability percentage was 67.7%, in patients with type-2 diabetes mellitus and 89.1% in patients with schizophrenia. Baseline GS along with other variables, also predicted changes in social functioning in major depressive disorder, bipolar disorder, and schizophrenia, with variability percentages of 67.3, 36, and 59%, respectively. Conclusion: GS combined with other variables significantly predicted changes in cognitive performance and social functioning in people with SMIs or type-2 diabetes mellitus. Interventions aimed to improve the overall physical conditions of patients who have poor GS could be a therapeutic option that confers positive effects on cognitive performance and social functioning.

## Introduction

Physical fitness, cognitive ability, and social functioning are critical for living a healthy and happy life. Although connections between these three health components have been suggested, the causality and directionality of these relationships have not yet been fully elucidated (Firth et al., 2018a). These components are impaired in elderly patients and in those who, regardless of age, have chronic diseases such as type-2 diabetes mellitus (T2DM), major depressive disorder (MDD), bipolar disorder (BD), and schizophrenia (SZ) (Guerra and Amaral, 2009; Catalá-López et al., 2013). Additionally, physical fitness, cognitive ability, and social functioning contribute to frailty. Traditionally, frailty has been defined as “a state of greater vulnerability to stressors, which is a consequence of the decrease in the physiological reserve in multiple organ systems, assuming a limited ability to maintain homeostasis” (Fried et al., 2001). Thus, understanding the relationships between these three components of frailty and the clinical implications of these relationships for people with chronic diseases, such as severe mental illnesses (SMIs) or T2DM, is critical.

In the pathology of SMIs or T2DM, frailty becomes evident with the progression of the disease. From the time of onset of these diseases, there are notable negative impacts in the work life, interpersonal relationships, or self-care of patients, compared to the premorbid phase of the disease. In this regard, understanding the progression of frailty is essential for assessing the deterioration in the quality of life. The causal relationship between the progression of mental pathologies/T2DM and frailty status is not clear, but previous research has suggested that they may have parallels (Rosas-Carrasco et al., 2011; Bartley et al., 2016). These diseases have been associated with reduced autonomy of patients and a potential decrease in physiological capacity and social functioning (Robertson et al., 2013; Rahman, 2018). Therefore, frailty contributes to the pathology of these comorbidities and could trigger a quicker, more progressive deterioration in the quality of life.

Physical capacity is a key component of frailty. Certain physical parameters, such as grip strength (GS), gait speed, and weight loss, may be measured to assess the frailty status of patients. Additionally, previous research has indicated that non-physical aspects, such as nutritional status, mental health, and changes in cognitive ability, could also contribute to frailty (Robertson et al., 2013). GS is a good indicator of frailty and could be useful as an indicator of pre-frailty status in patients with impaired GS (Alfaro-Acha et al., 2006; Boyle et al., 2009). Moreover, an increased GS is related to better performance of functional tasks, such as walking and getting up from a seated position. Additionally, GS affects the ability to perform self-care tasks (Goins et al., 2011; Robertson et al., 2013). In fact, GS has been suggested as a better marker of frailty than chronological age (Syddall et al., 2003; Guerra and Amaral, 2009; Ortega et al., 2012). During a 4-year follow-up, Leong et al. (2015) determined that reduced GS was related to an increase in all-cause mortality and, cardiovascular and non-cardiovascular mortality. The results indicated that GS was the best predictor of mortality, surpassing systolic blood pressure.

Cognitive frailty, which is defined as the deterioration of cognitive abilities that is associated with a state of frailty, is being recognized as a fundamental part of individual vulnerability and resilience to stressors (Panza et al., 2015). In addition, there is evidence of a pathophysiological relationship between the state of physical and cognitive frailty (Fritz et al., 2017). Previous research suggests that there is an association between physical frailty and decreased cognitive abilities; therefore, these two conditions may have similar mechanisms (Rockwood and Mitnitski, 2007; Robertson et al., 2013; Suo et al., 2016). However, the causal relationship between them is not clear (Debette and Markus, 2010). Therefore, it is necessary to elucidate the potential relationship between cognitive and physical frailty.

Furthermore, decreased GS has been associated with lower executive function, focus, working memory, language, semantic categorization, and general cognition in non-demented older people (Fritz et al., 2017). Recent research demonstrates that the decrease in GS at baseline is more strongly associated with the development of mild cognitive impairment and that higher GS at baseline protects cognitive function, functional status, mobility and mortality in people aged 60 years and older (Bohannon, 2008; Boyle et al., 2009; Rijk et al., 2016; Veronese et al., 2016). GS measured with a dynamometer is a reliable measure for estimating the frailty status of patients. Although other relevant components of frailty have been studied, muscle strength is a very simple non-invasive measure, and has been shown to be of remarkable importance as a marker of physical and cognitive deterioration.

Frailty can lead to the development of numerous chronic diseases, but can also be caused by multiple comorbidities (Vancampfort et al., 2019). Reportedly, SMIs are included as chronic diseases that are bi-directionally associated with frailty (Vetrano et al., 2018). For example, patients with SZ suffer from different comorbidities, some of which are related to reduced physical activity; these conditions include, reduced bone mass. On the other hand, these patients are treated with antipsychotics and other medications (Firth et al., 2018b). These factors contribute to an increased risk of adverse events and worsened overall health. In SMIs, GS and cognitive impairment have been found to be associated and cognitive performance is significantly correlated with physical health (Bohannon, 2015; Firth et al., 2018b; Hidese et al., 2018; Laredo-Aguilera et al., 2019; McGrath et al., 2019). Therefore, measuring GS could be a valid indicator of future cognitive performance and social functioning impairment in patients with SMIs.

To the best of our knowledge, no study has evaluated the association between GS, as a measure of frailty, and cognitive performance and its implications for social functioning in patients with SMIs and T2DM. Furthermore, no known studies have included patients with mental illnesses or T2DM. The present study aimed to investigate if GS is a significant predictor of changes in cognitive performance and social functioning after 1 year of follow-up and determine, if the relationships between GS, cognitive performance, and social functioning measures were different among the groups. Thus, we formulated the following objectives: a) To elucidate the relationship between the decrease in GS and cognitive performance and its implications on social functioning, b) To analyze the differences between motor, cognitive, and social variables in the different groups, and c) To examine whether there are predictive relations between a decrease in GS and impairment in cognitive performance or social functioning.

## Materials and Methods

### Study Design and Ethical Considerations

This article shows partial results of a more extensive study that seeks the identification and validation of peripheral biomarkers for a neurocognitive deficit in depression, BD, SZ, and T2DM. Only those variables that could provide clarity to the study of the GS as a measure of frailty were included in the analyses. Statistical data that did not represent significant differences were excluded. This prospective, comparative cohort study was conducted between April 2015 and January 2018. During this 1-year, follow-up study, several biomarkers, frailty components, and clinical, sociodemographic, and neurocognitive functioning data were collected at baseline (T1) and after 1 year (T2). The patient sample was recruited from mental health units (MHU) at several towns in the province of Valencia (Spain) (Foios, Catarroja, Paterna, and Sagunto), the psychiatry outpatient clinic and endocrinology department of the University Hospital Dr. Peset and in the MHU of the Health Center of Miguel Servet, in Valencia City. Healthy controls (HC) were residents of the same areas of the patients. We compared them in terms of sex, age, and years of education to the extent possible. Study procedures were explained to the participants and all participants provided informed consent. The ethical committees or an institutional review board at each participating center approved the study protocol, and the study was conducted in accordance with the ethical principles of the Declaration of Helsinki.

### Participants

At baseline (T1), the sample consisted of 165 subjects, including 30 patients with SZ, 42 patients with BD, 34 patients with MDD, 30 patients with T2DM, and 29 genetically unrelated HC. At T2, there were 125 subjects, including 27 patients with SZ, 29 patients with BD, 24 patients with MDD, 25 patients with T2DM, and 20 HC. SZ, BD, and MDD, were diagnosed according to the criteria of the Diagnostic and Statistical Manual of Mental Disorders – DSM-5 (American Psychiatric Association APA, 2014). T2DM was diagnosed based on the Standards of Care criteria of the American Diabetes Association (American Diabetes Association ADA, 2015). For recruitment as HC, the absence of physical illness, pharmacological treatments, and family history of SMI in first-degree relatives was required. In addition to being diagnosed with one of the above-mentioned conditions, the other inclusion criterion was the ability to understand and give written consent. For BD and MDD, it was necessary to meet the remission criteria of an acute affective episode, and patients with SZ had to be clinically stable. Patients with T2DM had to be free of severe diabetic neuropathy and kidney disease (serum creatinine <1.5 mg/dl). General exclusion criteria for all groups included: clinical conditions that hindered the study design, current hospitalization, documented cognitive impairment (intellectual disability or dementia), disability or inability that prevented understanding of the protocol, current substance abuse disorder, pregnancy, intake of steroids, corticosteroids, antioxidants, antibiotics, and immunologic therapies, fever over 38°C, and history of vaccination within 4 weeks of the evaluation. The same inclusion and exclusion criteria were used at T1 and T2. Patients with reduced mobility or motor deficits that made it difficult to perform or prevented them from performing the GS test were excluded from this study.

### Assessments

The assessments were conducted by the same experienced psychologists and psychiatrists of the research group. Sociodemographic data, including sex, age, years of education, and motor laterality, were collected at T1. For patients, the age of disease onset and illness duration were obtained and the body mass index (BMI; kg/m^2^) was measured for all the participants. The evaluations of each patient were carried out in the morning at their referral health centers, and were one or two hours in length with an intermediate break when necessary. Manual force was evaluated initially, followed by the remainder of the tests. The pharmacological treatment of each patient was recorded in detail and was taken into account as a covariate within the statistical analysis. All of the tests and scales were applied and scored according to the methodologies described in their respective manuals (see cited references below). To transform the direct scores into *Z* scores, a HC group, not genetically related to the patients, was used.

Clinical evaluations were conducted using the following scales: (i) the Hamilton Depression Rating Scale (HDRS) (Hamilton, 1960; Ramos-Brieva and Cordero-Villafáfila, 1986), (ii) the Young Mania Rating Scale (YMRS) (Young et al., 1978; Colom et al., 2002), (iii) the Positive and Negative Symptoms Scale (PANSS) (Peralta and Cuesta, 1994), which is also used to assess the severity of illness in psychiatric patients, and (iv) the Clinical Global Impression (CGI) scale (Vieta et al., 2002). The HDRS and YMRS are used for cases of BD and MDD that meet the remission criteria (Euthymia = HDRS < 9 and YMRS < 7).

Social functioning was evaluated using: (i) the Functional Assessment Short Test (FAST) (Rosa et al., 2007), (ii) the Short Form-36 Health Survey questionnaire (SF-36) (Alonso et al., 1995), and (iii) the Quality of Life of the World Health Organization assessment instrument (WHO-QoL-Bref) (Bobes et al., 2004).

Cognitive performance was evaluated using a battery of neurocognitive tests and subtests previously used by our group (Balanzá-Martínez et al., 2005; Tabarés-Seisdedos et al., 2008; Salazar-Fraile et al., 2009; Selva-Vera et al., 2010; Correa-Ghisays et al., 2017). Evaluation of cognitive performance was divided into (i) the premorbid Intelligence Quotient (IQ), which was calculated using the Wechsler Adult Intelligence Scale (WAIS-III) vocabulary subtest (Weschler, 1999), (ii) the Cognitive Reserve (CR), which was estimated based on the results of the WAIS-III Vocabulary subtest (Lyman, 1971; Weschler, 1999), considered a classical measure of the level of intelligence before the onset of a mental disorder, and calculated based on the number of years of formal education, and (iii) the Global Cognitive Score (GCS), which was calculated by averaging seven cognitive domain scores, including learning and verbal memory, cognitive flexibility, verbal fluency, working memory, short-term memory, visual memory and processing speed scores.

**FIGURE 1:**
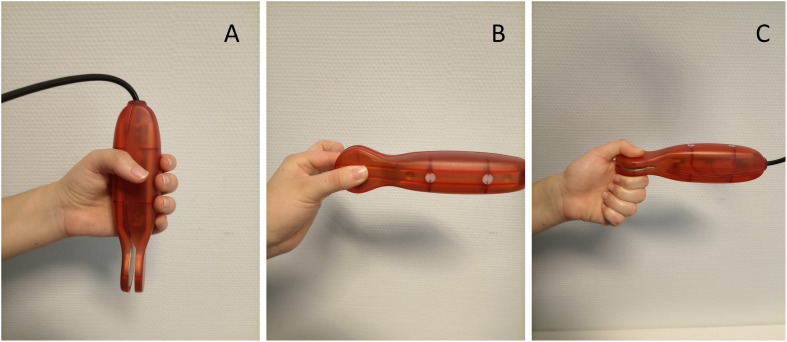
Electronic dynamometer (NedVEP/IBV) functional positions: **(A)** handgrip, **(B)** lateral/key pinch (thumb pad and lateral aspect of index finger), **(C)** tip pinch (thumb opposed by the index and long fingers).

GS was measured using an electronic dynamometer (NedVEP/IBV), with a built-in extensometric transducer and NedDiscapacidad/IBV software V4.1.1 from the Valencia Institute of Biomechanics (Lorenzo-Agudo et al., 2007; Hervás et al., 2011; Montero-Vilela et al., 2012). Each dynamometer was calibrated before every test for each participant. The test was performed with the participant sitting in an upright position in a chair with a backrest and without armrests. The feet had to be supported on the floor with 90° of knee flexion. The arm was positioned with 90° of elbow flexion and neutral pronosupination of the forearm (Su et al., 1994). The hand strength was recorded for three functional positions: (A) handgrip, (B) lateral/key pinch (thumb pad and lateral aspect of index finger), (C) tip pinch (thumb opposed by the index and long fingers), as previously described (Montero-Vilela et al., 2012; McQuillan et al., 2016) (Figure 1). For each functional position, three maximum strength scores (in N or kg) were obtained for both hands. The repetitions in each hand did not differ by more than 10% and the average was calculated for each side (Mathiowetz et al., 1984). To simplify the GS measures and inspect if only a frailty marker could be obtained to predict changes at T2, two global measures were created, such as the means of the following measures: (i) the Global Handgrip Score (GHGS) from the right and left handgrip (RHG and LHG, respectively), and (ii) the Global Pinch Score (GPS) from the right lateral/key pinch (RLP), left lateral/key pinch (LLP), right tip pinch (RTP), and left tip pinch (LTP).

**FIGURE 2:**
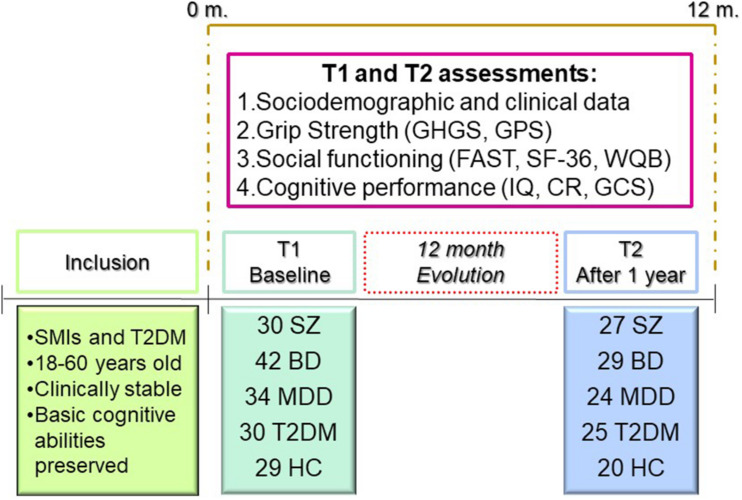
Research methodology.

Figure 2 illustrates the research methodology adopted in this study.

### Statistical Analyses

Data were analyzed using Statistical Package for Social Sciences (SPSS) version 24 for Windows (SPSS Inc., Chicago, IL, United States). Descriptive analyses were conducted using a one-way analysis of variance for continuous variables and the chi-squared test for categorical variables. Normality was assumed for all continuous variables because the sample is sufficiently representative of the target population, which was statistically verified. This fact guarantees that the variables are distributed in a normalized way. The differences between groups for the motor, cognitive, and social variables at T1 and T2 were assessed using a one-way analysis of covariance, with sex and age as co-variates. A *post hoc* analysis with Bonferroni corrected pairwise t-test and Mann–Whitney *U* tests were performed to examine the differences between groups. To test the predictive capacity of GS at baseline to explain the variance in cognitive performance and social functioning at T2, a linear regression analysis was performed using a predictive model that included only sociodemographic, clinical, social, and cognitive variables that were significant for each group. For all analyses, *p* < 0.05 was considered statistically significant. The procedure to create the predictive models was the following: first, a predictive analysis was performed only with the motor functioning variables; however, since they were not optimal by themselves, they were associated, one by one, to the sociodemographic, clinical, cognitive, and social variables. Then, the predictive models were generated including and combining the statistically more powerful variables; therefore, we obtained the optimal predictive combination. No more than five variables were included in each model, thus guaranteeing the correct performance of the analysis.

## Results

### Sample Description

The sociodemographic and clinical data of the five sample groups at T1 are shown in Table 1.

**TABLE 1:** Sociodemographic and clinical characteristics of the sample at T1.

**Variables^a^**	**HC**	**T2DM**	**MDD**	**BD**	**SZ**	***Statistical analyses***	
	**(*n* = 28)**	***(n* = 30)**	**(*n* = 35)**	**(*n* = 42)**	**(*n* = 30)**	**F(*p*)^*e*^**	***Post hoc* test^g^**
*Sociodemographic variables*							
Sex^*b,f,h*^	18(64%)	9(30%)	24(68%)	21(50%)	7(23%)	20.1****	SZ, T2DM < HC; SZ < BD SZ, T2DM < MDD
Age (years)	36.6(14.5)	57.3(9.3)	47.3(11.8)	50(9.5)	40.8(10.7)	15.3****	HC < SZ, MDD, BD, T2DM SZ < BD, T2DM MDD < T2DM
Years of Education	16.1(3.3)	12.5(5.8)	11.9(4.3)	11.6(4.4)	10.4(3.3)	7.1****	SZ, BD, MDD, T2DM < HC
Motor Laterality^c^	23(82%)	27(90%)	34(97%)	38(90%)	28(93%)	NS	
*Clinical variables*							
HDRS^d^	2.0(1.8)	3.9(3.9)	11.6(8.3)	6.4(4.4)	7.0(5.8)	14.2****	HC < BD, SZ, MDD T2DM, BD, SZ < MDD
YMRS^d^	0.8(1.6)	1.5(2.2)	1.9(2.6)	3.5(4.5)	3.2(4.9)	3.4**	HC < BD
PANSS positive^d^	7.0(0.0)	7.0(0.0)	7.0(0.3)	8.5(3.8)	10.6(4.3)	10.6****	HC, T2DM, MDD, BD < SZ
PANSS negative^d^	7.0(0.0)	7.1(0.7)	8.4(4.9)	10.3(6.5)	18.6(10.1)	20.1****	HC, T2DM, MDD, BD < SZ
PANSS general^d^	16.0(0.0)	17.0(2.3)	19.8(8.6)	22.7(9.9)	31.8(12.7)	16.9****	HC < BD, SZ T2DM, MDD, BD < SZ
PANSS total^d^	30.0(0.0)	31.2(2.8)	35.4(13.4)	41.6(18.9)	61.1(24.4)	20.2****	HC < BD, SZ T2DM, MDD, BD < SZ
CGI^d^	1.0(0.0)	1.9(1.0)	3.3(1.2)	3.5(0.7)	4.5(1.0)	63.8****	HC < T2DM, MDD, BD, SZ T2DM < MDD, BD, SZ MDD, BD < SZ
Age of onset (years)	-	44.3(9.8)	35.3(12.1)	26.5(8.6)	25.6(8.0)	16.8****	SZ < MDD, T2DM BD < MDD, T2DM MDD < T2DM
Illness duration (years)	-	13.0(9.0)	12.0(11.6)	23.4(11.5)	15.2(8.4)	25.9****	MDD, T2DM, SZ < BD
BMI (kg/m^2^)	24.9(5.1)	30.4(4.3)	28.6(5.8)	29.7(5.6)	31.9(5.4)	7.0****	HC < BD, T2DM,SZ

^a^ Expressed as mean(standard deviation) except when indicated, ^b^ female n(%), ^c^ right-handed n(%). ^d^ Lower scores represent a better outcome. ^e^ ANOVA. ^f^ Chi-squared test. ^g^ Bonferroni test. ^h^ Mann–Whitney U test. Abbreviations: HC = Healthy control, T2DM = Type-2 Diabetes Mellitus, MDD = Major Depressive Disorder, BD = Bipolar Disorder, SZ = Schizophrenia, HDRS = Hamilton Rating Scale for Depression, YMRS = Young Mania Rating Scale, PANSS = Positive and Negative Syndrome Scale, CGI = Clinical global impression, BMI = Body Mass Index, ANOVA = Analysis of variance, NS = not significant. (NS = *p* > 0.05; *p ≤ 0.05; **p ≤ 0.01; ***p ≤ 0.001; ****p ≤ 0.0001).

#### Sociodemographic Variables

The average age of the HC group was significantly lower than the rest of the groups. SZ patients had the lowest mean age, while T2DM patients had the highest mean age; there were significant differences between the groups. The years of education was significantly different, with participants in the HC group having the most. The years of education were similar among the clinical groups. No significant differences were found in motor laterality.

#### Clinical Variables

There were significant differences in the scores of the PANSS tests (negative, positive, general, and total), with SZ patients having the highest scores, compared to the other groups. Patients with BD had higher scores in the total and general PANSS compared to the HC group. Scores on the HDRS were higher in patients with MDD, BD, and SZ compared to HC. Among them, patients with MDD showed higher scores than those with SZ, T2DM, and BD. Significant differences were also found for SZ, BD, MDD, and T2DM patients in terms of the CGI; patients with SZ had the worst scores. In addition, BMI was significantly higher in the SZ, BD and T2DM groups compared to the HC group.

### Between-Group Comparison of GS, Cognitive Performance, and Social Functioning

Grip strength measures, cognitive, and social variables of the five groups from the sample at T1 and T2 are shown in Table 2. Regarding the results obtained when comparing the motor variables between the clinical groups and HC, all of them showed significant differences at both T1 and T2. Notably, the GHGS and GPS indicated that the five groups had significantly different global GS measures. We observed significant differences in the GS, global GHGS, and GPS scores, for both hands. At T1, the MDD group performed significantly lower on the GHGS than the other three clinical groups. This difference remained at T2, but was only significant when compared to patients with T2DM. The GPS was also lower for MDD patients compared to T2DM and SZ patients at T1 and T2. For both time points, patients with SZ achieved significantly higher GPS scores than HC.

**TABLE 2:** Outcomes variables at Time 1 and Time 2 (*Z*-scores).

	**HC**		**T2DM**		**MDD**		**BD**		**SZ**		***Statistical analyses***			
	**T1 (*n* = 28)**	**T2 (*n* = 19)**	**T1 *(n* = 30)**	**T2 (*n* = 25)**	**T1 (*n* = 35)**	**T2 (*n* = 25)**	**T1 (*n* = 42)**	**T2 (*n* = 29)**	**T1 (*n* = 30)**	**T2 (*n* = 27)**	**T1 F(*p*)^*b*^**	***Post hoc* test^*d*^**	**T2 F(*p*)^*b*^**	***Post hoc* test^*d*^**
GS measures														
RHG	0.0(1.0)	−0.1(0.8)	0.2(1.0)	0.1(1.1)	−0.5(0.9)	−0.7(0.7)	−0.2(1.0)	−0.5(0.9)	0.0(0.9)	−0.2(0.8)	4.0**	MDD < SZ, T2DM	3.8**	MDD < T2DM
LHG	0.0(1.0)	−0.2(0.9)	0.2(1.1)	0.2(1.1)	−0.5(1.0)	−0.8(0.9)	−0.1(1.1)	−0.3(1.0)	0.0(1.0)	−0.2(0.9)	4.1**	MDD < BD, T2DM	4.7***	MDD < BD, T2DM
GHGS	0.0(1.0)	−0.2(0.8)	0.2(1.1)	0.2(1.1)	−0.6(1.0)	−0.8(0.8)	−0.1(1.1)	−0.4(0.9)	0.0(1.0)	−0.2(0.9)	4.2**	MDD < BD, SZ, T2DM	4.3**	MDD < T2DM
RLP	0.0(1.0)	0.3(0.8)	0.4(0.9)	0.7(0.7)	−0.1(0.8)	0.2(0.7)	0.1(0.9)	0.2(0.7)	0.7(0.6)	0.8(0.6)	5.7****	MDD < T2DM, SZ; HC < SZ	4.8***	MDD, HC < SZ
LLP	0.0(1.0)	0.3(0.8)	0.6(1.1)	0.9(0.9)	−0.1(0.9)	0.3(0.8)	0.3(1.0)	0.5(0.9)	0.9(0.7)	0.9(0.7)	6.3****	HC, MDD < T2DM, SZ	4.2**	MDD, HC < SZ
RTP	0.0(1.0)	0.2(0.6)	0.4(1.1)	0.8(0.9)	−0.3(0.9)	0.0(0.8)	0.1(1.1)	0.3(1.0)	0.6(0.6)	0.5(0.7)	5.0***	MDD < T2DM, SZ	3.6**	MDD < T2DM
LTP	0.0(1.0)	0.2(0.8)	0.6(1.2)	1.0(1.0)	−0.2(1.0)	0.0(0.8)	0.2(1.2)	0.3(1.2)	0.8(0.8)	0.7(0.9)	6.2****	HC, MDD < T2DM, SZ	4.9***	MDD, HC < T2DM
GPS	0.0(1.0)	0.3(0.7)	0.5(1.1)	0.9(0.9)	−0.2(0.9)	0.1(0.7)	0.2(1.1)	0.4(0.9)	0.8(0.7)	0.8(0.7)	6.3****	MDD < T2DM, SZ; HC < SZ	4.9***	MDD < SZ, T2DM; HC < SZ
Social functioning														
FAST	0.0(1.0)	0.0(0.8)	−1.2(1.8)	−1.0(1.5)	−3.7(2.6)	−3.5(2.5)	−4.3(1.9)	−3.9(1.9)	−5.6(2.4)	−4.7(2.5)	22.3****	SZ < BD, MDD, T2DM, HC BD, MDD < T2DM, HC	9.7****	SZ, BD, MDD < T2DM SZ, BD, MDD < HC
SF-36	0.0(1.0)	0.2(0.5)	−1.2(1.8)	−1.0(1.7)	−3.9(2.1)	−3.8(2.7)	−2.6(1.9)	−2.6(1.8)	−1.9(1.9)	−2.3(1.9)	14.4****	MDD < BD, SZ, T2DM, HC BD, SZ < HC	9.1****	MDD < BD, SZ, T2DM, HC
WQB	0.0(1.0)	0.3(1.1)	−0.6(1.2)	−0.5(1.4)	−2.2(1.2)	−2.2(1.8)	−1.6(1.2)	−1.9(1.1)	−1.3(1.0)	−1.2(1.2)	13.7****	MDD < SZ, T2DM, HC BD, SZ < HC	7.8****	MDD < SZ, T2DM, HC
Cognitive performance														
IQ	0.0(1.0)	1.5(1.0)	−0.2(1.2)	0.7(1.2)	0.0(1.3)	1.0(1.1)	0.0(1.3)	0.1(1.4)	−1.0(1.5)	0.1(1.1)	3.4**	SZ < T2DM, MDD, BD	4.7***	BD,SZ < HC
CR^*a,c,e*^	6(21%)	2(10%)	16(53%)	11(44%)	16(46%)	12(48%)	23(55%)	18(62%)	22(73%)	20(74%)	16.4**	HC < T2DM, MDD, BD, SZ MDD < SZ	20.1****	HC < T2DM,MDD, BD, SZ MDD, T2DM < SZ
GCS	0.0(0.5)	0.3(0.6)	−0.9(0.8)	−0.8(0.9)	−0.8(0.8)	−0.7(0.9)	−1.3(1.0)	−1.1(0.9)	−1.7(1.0)	−1.5(0.9)	16.6****	SZ < BD, T2DM, MDD, HC BD < T2DM, HC	14.6****	SZ < BD, T2DM,MDD,HC BD, MDD < HC

Expressed as mean (standard deviation) except when indicated.^*a*^Low *n*(%).^*b*^ANCOVA.^*c*^Chi-squared test.^*d*^Bonferroni test.^*e*^Mann–Whitney *U* test.HC, healthy control; T2DM, type-2 diabetes mellitus; MDD, major depressive disorder; BD, bipolar disorder; SZ, schizophrenia; T1, Time 1; T2, Time 2; RHG, right handgrip; LHG, left handgrip; GHGS, Global Handgrip Score; RLP, right lateral/key pinch; LLP, left lateral/key pinch; RTP, right tip pinch; LTP, left tip pinch; GPS, Global Pinch Score; WQB, WHO-QoL-BREF; IQ, Intelligence Quotient; CR, cognitive reserve; GCS, Global Cognitive Score; ANCOVA, analysis of covariance; SF-36, Short-form 36; FAST, Functional Assessment Short Test; NS, not significant.^*NS*^*p* > 0.05; *p ≤ 0.05; **p ≤ 0.01; ***p ≤ 0.001; and ****p ≤ 0.0001.

Regarding social functioning, patients with SZ had significantly lower FAST total scores than the rest of the groups at T1. Moreover, the BD and MDD groups had worse results than the T2DM and HC groups. At T2, the SZ, BD, and MDD groups showed significantly lower FAST total scores compared to the T2DM and HC groups. The SF-36 test revealed that patients with MDD had the lowest scores, at T1 and T2. In addition, the SZ and BD groups obtained lower scores than the HC at T1; a similar outcome was observed with the WHO-QoL-Bref. The rest of the results for WHO-QoL-Bref were similar to those of the SF-36 for all groups, except the BD group, with worse results in the MDD group at T1 and T2.

For the analysis of cognitive performance, different variables were analyzed; among them, we highlight the GCS. The IQ, CR, and GCS were significantly different among the five groups (*p* < 0.001). *Post hoc* analyses revealed that the GCS was significantly more affected in the SZ group, with the worst scores at both time points. Likewise, the BD group had lower scores at T1 compared to the HC and T2DM groups. Similarly, this occurred at T2 in patients with BD and MDD, but only when compared to the HC group. The differences in performance between the time points within each group were not significant.

### Results of the Predictive Model

Table 3 shows the results of the statistical analysis from the relative contributions of the factors studied at baseline (T1), to explain the variation of the results after 1 year of follow-up (T2). In each of the groups, different variables have been included that could explain the endpoint performance at T2. We have observed that the GHGS and/or the GPS alone did not give significant results, in terms of their ability to predict cognitive or social functioning. In contrast, with other combinations that considered more specific motor domains (not just the global one), and other cognitive domains, we found that they were predictive of the results at T2. Therefore, for each of the groups included in the study, combinations of different variables were analyzed together with motor variables to determine if any of them had predictive value, in terms of changes in cognitive performance and social functioning. The results of each of the groups were as follows:

**TABLE 3:** Predictive model.

**Dependent variables at T2**	**Predictors at T1 associated**	**β**	**95% CI**	***t***	**Percent of variance explained (adjusted *R*^2^)**
Group: T2DM					
GCS	CR	−0.557	−1.46 to −0.53	−4.43****	67.7
	Illness duration	−0.293	−0.05 to 0.00	−2.01*	
	RHG	0.309	0.00 to 0.05	2.11*	
Group: MDD					
SF-36	CGI	−0.567	−1.65 to −0.59	−4.38****	67.3
	BMI	−0.324	−0.27 to −0.02	−2.48*	
	LHG	0.301	0.01 to 0.16	2.37*	
Group: BD					
FAST	PANSS-T	−0.430	−0.06 to −0.01	−2.73**	35.9
	RHG	0.410	0.01 to 0.13	2.60**	
WQB	PANSS-N	−0.436	−0.11 to −0.01	−2.79**	36.7
	RTP	0.413	0.03 to 0.30	2.64**	
Group: SZ					
GCS	GCS	0.837	0.66 to 0.95	11.55****	89.1
	Motor laterality	−0.235	−1.97 to -0.43	−3.23***	
	RHG	0.086	−0.00 to 0.02	1.24*	
SF-36	FAST	0.595	0.26 to 0.71	4.40****	59.0
	RHG	0.357	0.16 to 1.33	2.64**	

T1, Time 1; T2, Time 2; CI, confidence interval; T2DM, type-2 diabetes mellitus; MDD, major depressive disorder; BD, bipolar disorder; SZ, schizophrenia; RHG, right handgrip; LHG, left handgrip; RTP, right tip pinch; WQB, WHO-QoL-BREF; PANSS, Positive and Negative Syndrome Scale; CGI, clinical global impression; BMI, body mass index; CR, cognitive reserve; GCS, Global Cognitive Score; SF-36, Short-form 36; FAST, Functional Assessment Short Test; NS, not significant.^*NS*^*p* > 0.05; *p ≤ 0.05; **p ≤ 0.01; ***p ≤ 0.001; and ****p ≤ 0.0001.

In the SZ group, 89% of the variance of the GCS can be explained after 1 year, when considering the changes that have been produced in the RHG, the motor laterality, and the GCS from T1. In this group, with these combinations, the highest percentage of variability has been explained in terms of changes in cognition. Regarding the changes in the social functioning, as evaluated with SF-36, the FAST with the RHG explained up to 59% of the changes in the results. This indicates that, in patients with SZ, RHG may have a predictive component, in terms of cognitive performance and social functioning. In contrast, patients with BD obtained the lowest percentages of variance. In this case, social functioning, which was evaluated with FAST and WHO-QoL-Bref, was explained by approximately 36% due to changes in PANSS and GS, measured in one case with RHG (for FAST) and in another with RTP (for the WHO-QoL-Bref). Those with MDD are affected by their social functioning capacity, which was measured with the SF-36, at a level of 67.3%, when considering BMI, CGI, and LHG. We have highlighted that LHG has influenced the predictive results at T2 in these patients; however, 97% of the patients in the MDD group were right-handed, so the strength of the left hand cannot be explained, in the case that has intervened in the variability of the results after one year. We conclude that changes in GS, BMI and overall clinical impression affect the functional ability of patients with MDD. Patients with T2DM are affected by their cognitive functioning through the GCS; if we combine the variables of CR, illness duration, and RHG, cognitive functioning was explained by up to 67.7%. These results indicate that in our group, changes in GS, along with years of illness and CR, could act by predicting deterioration in global cognition over a 1 year period.

## Discussion

The purpose of this study was to determine the clinical implications of GS, in regard to cognitive performance and social functioning, in patients with SZ, BD, MDD and T2DM, and confirm if GS can provide valuable information about physical function, which may be considered a frailty marker. Our findings indicate that GS can, in part, account for variabilities in cognitive and social functioning after one year of follow-up. However, it is clear that along with other variables, changes in physical performance influence long-term and predict cognitive and social functioning impairment in patients with MDD, BD, SZ, and T2DM, when compared to HC. In our study, changes in GS significantly influenced the GCS and we emphasized that the RHG is the most powerful motor variable, as it contributed to the most changes after 1 year. These findings are consistent with the literature, which indicates that the best results are obtained when the task is performed in the most comfortable position and with the right hand (Sternäng et al., 2014; Soysal et al., 2017; Firth et al., 2018a; Smith et al., 2018). Furthermore, we observed that the relationships between GS and, cognitive and social functioning measures were different for people with T2DM, MDD, BD and SZ. Each of the combinations of variables, which are different for each group, can explain the variability in the results after one year of follow-up. In the case of patients with T2DM, CR and illness duration together with RHG are fundamental for the cognitive impairment, accounting for almost 70% of the variability at T2. In this regard, another study found that diabetic microangiopathy and/or chronic inflammation in these patients, which is closely related to T2DM pathology, could be related to a deterioration of physical abilities, as evidenced by a decrease in GS (Zilliox et al., 2016). Similarly, previous research asserts that a worsened clinical status of MDD patients is associated with a decrease in physical activity (Montero-Vilela et al., 2012). In addition, changes in diet, weight, or BMI of MDD patients, can result in lower GS (Vancampfort et al., 2011). In this study, those changes have been shown to have a long-term impact on the social functioning of the MDD patients. Smith et al. (2018) reached the same conclusion after analyzing GS in patients with depression and overweightness. The findings of our study demonstrate, according to previous literature, that a weaker GS is associated with a lower quality of life; in turn, a low quality of life has a detrimental impact on mental health (Whiteford et al., 2015). These changes may explain the 67.3% variability in the social functioning for patients with MDD; despite the inclusion of BMI for the different groups, all clinical groups are equal and only the HC group has significant differences in BMI compared to the other groups.

However, studies regarding GS in BD patients are scarce. Firth et al. (2018b) demonstrated that GS predicted cognitive impairment in these patients. In our study, changes in the total and negative PANSS together with GS (RHG and RTP) predicted changes in social functioning. Symptom worsering, such as changes in appetite, smoking and/or drinking alcohol, sleep disorders, reductions in physical activity, and changes in body composition and metabolism, influenced the risk of decreased physical functions, which determine a greater degree of difficulty of the patient in terms of autonomy, work performance, and social functioning (up to 36% in our study) (Fried et al., 2001). However, more variability was observed in the SZ group. Almost 90% of the changes in the GCS at T2 are explained by the RHG, along with the motor laterality and the GCS at T1. It is noteworthy that, in this group, the GCS was significantly lower than that of the rest of the groups, and this was maintained at both time points. Similarly, patients with SZ showed lower scores in the social functioning at T1 compared to the other groups; the scores were worse than those of the HC and T2DM groups at T2. RHG, motor laterality, and GCS were the best predictors of changes in cognitive performance after 1 year in SZ patients (89.1%); this was similar to the ability of RHG and FAST to predict impairment in social functioning, when measured with the SF-36 (59%).

**FIGURE 3:**
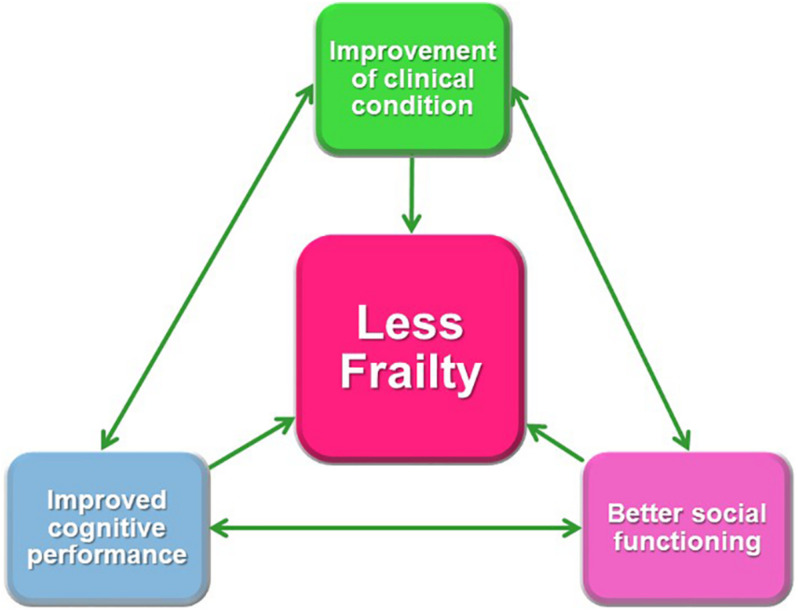
Relationship between frailty (grip strength), cognitive performance, and social functioning.

Therefore, the measurement of GS (and other variables) in the psychiatric population and in patients with T2DM could be a valid indicator to predict cognitive and social impairment in the future. Thus, GS does not only influence cognitive and social changes since the physical condition in these patients is closely associated with the state of their disease. These findings suggest that people who are physically weaker or those whose physical abilities are diminished may be more vulnerable to a worsened pathology, that is, they may be in a state of physical and/or cognitive frailty. It is difficult to biologically explain this situation, but the reverse causality could partially explain it and, some of the results of the study: people with SMI may be less likely to participate in any social activity, including physical activities, which would result in a lower physical condition and lower GS due to inactivity. On the other hand, psychiatric disorders are highly related to maladjustment of those who suffer from it; therefore, their social functioning is affected as soon as their disease worsens. As previously suggested, this could be explained because there is bidirectionality and/or causality of a third factor or factors in this relationship. The relationship between frailty (GS), cognitive performance, and social functioning is depicted in Figure 3.

Considering the GS as a marker of cognitive and social functioning, we could conclude that the increase of the GS as a measure of frailty combined with other variables such as the BMI, cognitive reserve, or disease duration could become both a new study objective and a therapeutic target to improve the cognitive and social functioning of people with SMI and DMT2. We propose that future research on the treatment of these diseases could explore the potential benefits of including strength training along with traditional psychotherapeutic and pharmacological interventions.

### Limitations

This study has some limitations that should be addressed. First, this study includes a small sample size (*n* = 165). Therefore, studies with larger sample sizes could provide more generalizable results. Additionally, after one year of follow-up, 40 patients were lost for different reasons, which resulted in a smaller sample at T2. Furthermore, a longer follow-up period should be included in future studies in order to detect stronger differences in the neurocognitive decline. Another limitation of the study is the average age of the participants (45 years). Because of this limitation, the results cannot be extrapolated to younger patients. Despite these limitations, this study is the first known study to investigate the association between GS and, cognitive and social functioning in patients with SZ, BD, MDD, and T2DM.

### Conclusions and Future Directions

Grip strength, especially RHG, plays a significant role in predicting changes in cognitive performance and social functioning in people with SZ, BD, MDD, and T2DM. There are differences between the studied groups in terms of variability of results and the variables included in the regression models, with GS included at T1 to explain changes over time (T2). RHG combined with other variables, which are different for each group, shows significant differences that may predict cognitive performance and social functioning during an 1-year follow-up. Therefore, it is clear that there is a common denominator (physical status), which is evidenced by the influence of GS on cognition and social functioning.

The results of this study are supported by the review of the medical literature where GS, when used as a representative parameter of frailty, is considered as a good biomarker of future neurocognitive and social changes. The variables taken into account in this study, and their functional implications within the state of frailty and cognitive deterioration in SMIs and T2DM have not been found in previous work. In our study, we found that, together with GS, some of these variables may have strong predictive values. Nonetheless, more studies should be conducted to further explore how and why these variables predict patient alterations over time.

Therefore, GS could be used for monitoring these patients, detecting changes in their physical condition that serve to intervene clinically, and preventing future adverse events. Future research should focus on establishing interventions that can be used to improve GS, cognitive status, and long term social functioning in patients who are in a state of frailty or pre-frailty. Interventions aimed to improve the overall physical conditions of patients who have poor GS could be a therapeutic option that confers positive effects on cognitive performance and social functioning.

Finally, we recommend carrying out additional studies, similar to this study, which include young people with chronic diseases such as severe early onset mental disorders and type 2 diabetes mellitus. This could expand the minimum and maximum reference values of GS as a marker of frailty. In addition, longitudinal studies at 5, 10, 15, 20, or more years of follow-up would be beneficial.

## Data Availability Statement

The datasets generated for this study are available on request to the corresponding author.

## Ethics Statement

The studies involving human participants were reviewed and approved by Comité Ético de Investigación Clínica del Hospital Clínico Universitario de Valencia. The patients/participants provided their written informed consent to participate in this study.

## Author Contributions

MA-D: interpretation of the data and drafting the manuscript. JS-O: statistical analysis, drafting the manuscript, and critical review of the manuscript. PC-G: study supervision, patient inclusion, acquisition and interpretation of data, drafting the manuscript, and critical review of the manuscript. JV-F: statistical analysis. VB-M: patient inclusion and critical review of the manuscript. GS-V: patient inclusion. PC-E, JF-M, CS-MV, MM-P, RA-A, MR-V, and BC-F: critical review of the manuscript. RT-S: study concept and design, study supervision, and critical review of the manuscript. All authors approved the final version of the manuscript.

## Conflict of Interest

The authors declare that the research was conducted in the absence of any commercial or financial relationships that could be construed as a potential conflict of interest.
